# Inverted Ultrathin Organic Solar Cells with a Quasi-Grating Structure for Efficient Carrier Collection and Dip-less Visible Optical Absorption

**DOI:** 10.1038/srep21784

**Published:** 2016-02-23

**Authors:** Sungjun In, Namkyoo Park

**Affiliations:** 1 Photonic Systems Laboratory, School of EECS, Seoul National University, Seoul 151-744, Korea

## Abstract

We propose a metallic-particle-based two-dimensional quasi-grating structure for application to an organic solar cell. With the use of oblate spheroidal nanoparticles in contact with an anode of *inverted, ultrathin* organic solar cells (OSCs), the quasi-grating structure offers strong hybridization between localized surface plasmons and plasmonic gap modes leading to broadband (300~800 nm) and uniform (average ~90%) optical absorption spectra. Both strong optical enhancement in extreme confinement within the active layer (90 nm) and improved hole collection are thus realized. A coupled optical-electrical multi-physics optimization shows a large (~33%) enhancement in the optical absorption (corresponding to an absorption efficiency of ~47%, AM1.5G weighted, visible) when compared to a control OSC without the quasi-grating structure. That translates into a significant electrical performance gain of ~22% in short circuit current and ~15% in the power conversion efficiency (PCE), leading to an energy conversion efficiency (~6%) which is comparable to that of optically-thick inverted OSCs (3–7%). Detailed analysis on the influences of mode hybridization to optical field distributions, exciton generation rate, charge carrier collection efficiency and electrical conversion efficiency is provided, to offer an integrated understanding on the coupled optical-electrical optimization of ultrathin OSCs.

Plasmonic effects offer powerful platforms for strong light scattering, extreme subwavelength confinement and extraordinary optical responses. By utilizing localized surface plasmons (LSP), plasmonic scattering, surface plasmon polaritons (SPP) and their combinations, the advantages of plasmonic effects have been employed in various applications such as antennas^1^,^2^,^3^, photodetectors^4^,^5^,^6^ and light harvesting devices^7^,^8^,^9^. Plasmonic effects are particularly effective and physically relevant for efficient light harvesting in organic solar cells, where the reduction of the active layer thickness is important. Because of the extremely short carrier (especially hole) diffusion length of organic materials, the electrical-thickness of the active layer needs to be strictly limited, to achieve efficient charge collection^7^,^10^. However, at the current stage of development, the active layer thickness in conventional OSCs is usually larger than the typical electrical scale length of carrier diffusion (<100 nm)^11^,^12^,^13^,^14^,^15^, to avoid the penalty of low optical absorption in organic photoactive materials.

Accordingly, past approaches at improving the performance of OSCs have been sought from mainly two different directions: electrically, by employing an inverted device to achieve efficient hole collection and stable anodic behavior^14^,^15^; and also optically, by using plasmonic nanoparticles, gratings, and nanostructures for efficient light trapping, confinement, and optical path length increases in the active layer^7^,^8^,^9^,^10^,^11^,^12^,^13^,^14^,^15^. Most notable recent developments include reports on enhancement of the PCE, through a combination of electrical and optical approaches; integrating plasmonic structures in the inverted device configuration^13^,^14^,^15^. However, mostly focusing on the experimental demonstrations, the full optimization considering the interplay between optical-absorption and electrical carrier collection has not been achieved. Most importantly, with the use of electrically-thick active layers (>100 nm) from the difficulty in obtaining strong, broadband optical mode in extreme confinement, efficient light harvesting from the optical toward the electrical forms has not thus far been fully executed^11^,^12^,^13^.

In this work, we propose a two-dimensional quasi-grating structure which provides both enhanced hole collection and broadband light absorption in tight mode confinement, for an inverted, ultrathin organic solar cell. Specifically, with the use of an oblate nanoparticle (ONP) array in electrical contact with the anode, we achieve short-distance, efficient hole collection due to the increased ONP-anode surface, in addition to dip-less wideband optical absorption from the strong hybridization between the LSP and plasmonic gap modes. Rigorous optical-electrical coupled numerical analysis shows that the large mode overlap between the LSP and gap modes, derived from the tight confinement of the light around the ONP-anode interface in the ultra-thin (90 nm) active layer, plays a crucial role in obtaining broadband (300~800 nm) and uniform (average ~90%) optical absorption spectra. With the introduction of the ONP array optimized in terms of both optical absorption and electrical collection, a large improvement of ∼33% in the optical absorption (corresponding to an absorption efficiency of ~47%, AM1.5G, visible) and significant electrical performance gains of ~22% in short circuit current and ~15% in PCE are demonstrated, when compared to control OSCs.

## Results

### Structure of inverted ultrathin OSCs with the proposed quasi-grating anode

**Figure 1:**
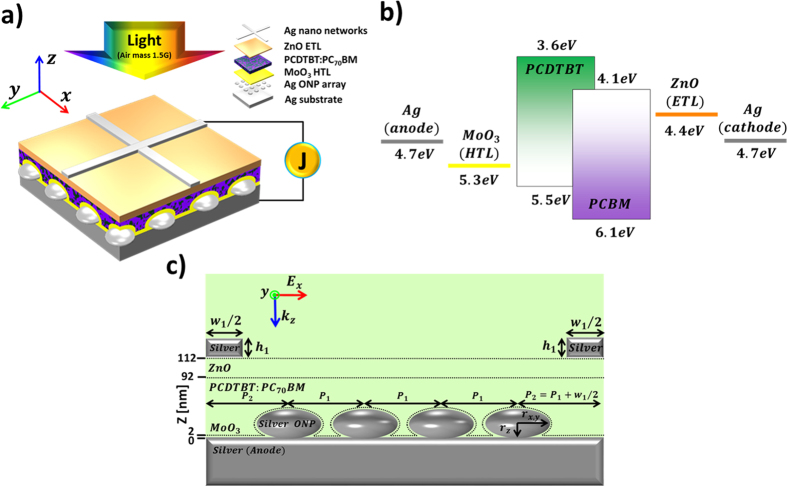
Quasi-grating inverted ultrathin OSCs. (**a**) Schematic perspective view of the quasi-grating inverted ultrathin OSCs with silver oblate nanoparticle array on silver substrate as an anode. (**b**) Energy level diagram of the materials used in the OSC. (**c**) The unit cell schematic of the *x*-*z* plane. The geometric parameters are: *P*_*1*_ = 200 nm, *P*_*2*_ = 230 nm ( = *P*_*1*_ + *w*_*1*_*/2*), *r*_*x*_ = *r*_*y*_ = 70 nm, *r*_*z*_ = 30 nm, *h*_*1*_ = 20 nm and *w*_*1*_ = 60 nm.

Figure 1 illustrates the proposed quasi-grating structure embedded in the inverted ultrathin OSCs. A control device assumes the same material compositions except for ONP array. The control OSC is composed of a 90 nm thick PCDTBT:PC70BM active layer, sandwiched between a 20 nm thick sol-gel processed ZnO electron-transport top layer and a 2 nm thick MoO3 hole-transport layer^16^,^17^ which sits atop a bulk Ag substrate (anode). The energy levels of these relevant functional layers are also shown in Fig. 1b. This alignment of the material compositions^15^,^18^,^19^,^20^,^21^,^22^ facilitates efficient electron transport between the electron transport layer (ETL) and the active layer, and allows extraction of holes without excessive interface recombination between the hole transport layer (HTL) and active layer. The use of molybdenum oxides as a HTL increases the work function of the anode, for better alignment to the energy level of active materials^15^,^18^. The periodic two-dimensional Ag nanowire network on the device surface serves as an electrical contact (cathode). Networks of Ag nanowire are made with a height of 20 nm, width of 60 nm and pitch of 860 nm, for sufficient light transmission (average transmission of 91.7%) and also to guarantee low sheet resistance (19.1 Ω/sq). Within the visible wavelength range of our interest, the designed transparent electrode provides comparable sheet resistance and higher optical transmittance, when compared to the conventional ITO electrodes (80~150 nm thickness)^23^,^24^. Optimization procedures for the transparent electrode are detailed in the Supporting Information (Figure S1). Within these frameworks, we load the ONP array in contact with the Ag substrate to form a quasi-grating anode structure, which is covered with an ONP-conforming MoO3 layer and a thin active layer, completing the flat top surface (Fig. 1c). It is noted that the choice of oblate nanoparticles in an ultrathin active layer yields optically superior properties when compared to the use of a spherical nanoparticle^7^, such as; enhanced in-plane scattering (Figure S2a), bigger modal volume and stronger light confinement (Figure S2b,c) all from the ‘oblate geometry’, and a larger surface area leading to greater scattering cross-section (Figure S2d). As noted earlier, with the increased anode surface area from the presence of oblate spheroid metallic particles dispersed within the active layer, in contact with the anode, enhancement in hole collection and reduced hole traveling lengths also become possible.

Considering the interplay between optical-absorption and electrical carrier collection, an optical-electrical multidisciplinary numerical optimization of the overall performance of the OSC has been carried out. First, the radius and aspect ratio of the ONP and the period of the ONP array have been optimized using finite element method (FEM) calculations, to obtain a structure that provides the largest optical absorption enhancement (weighted by AM1.5G). Then, considering the electrical performance contribution from the electrical carrier collection efficiency, the short circuit current and final ultimate performance factor (the PEC of the OSC) were calculated to find the optical-electrical optimum point (*r*_*x,y*_ = 70 nm, *r*_*z*_ = 30 nm and *P*_*1*_ = 200 nm), which involved scanning over a number of different quasi-grating parameter sets near the optically-obtained optimum structure (*r*_*x,y*_ = 90 nm, *r*_*z*_ = 30 nm and *P*_*1*_ = 200 nm).

### Optical characteristics: Dip-less broadband, wide angle absorption from strong mode hybridization

**Figure 2:**
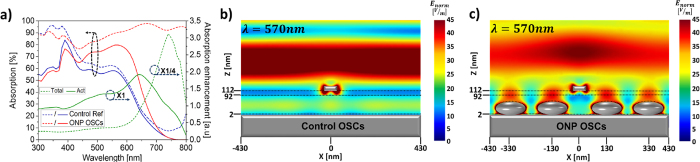
Optical properties of the proposed quasi-grating OSCs. (**a**) The optical absorption spectra and enhancement ratio of optical absorption relative to that of the control OSCs in the active layer (solid lines) and total structure (dotted lines): ONP array absent control OSCs (blue), quasi-grating inverted ultrathin OSCs (red; *P*_*1*_ = 200 nm, *r*_*x*_ = *r*_*y*_ = 70 nm, *r*_*z*_ = 30 nm) and absorption enhancement (green). Calculated electric field distributions (|E|) of (**b**) control reference case, and (**c**) optimized ONP case at *λ* = 570 nm.

Figure 2a shows the percentage optical absorption (i.e., the ratio of number of photons absorbed to number of incident photons at a given wavelength) in the active layer (solid lines) and total structure (dotted lines), calculated for the optimized OSC structure when illuminated by normal incident light (AM1.5G solar spectrum). The enhancement ratio of optical absorption relative to that of the control OSCs is also plotted in Fig. 2a. With the introduction of the ONP quasi-grating, the absorption in the active layer is considerably enhanced when compared to the control OSC, especially in the region of 430 ~ 740 nm, achieving dip-less total absorption (average ~90%) over the entire spectra. The cumulative improvements in optical absorption are found to be ~114% (total) and ~33% (active layer), respectively, from the definition of FOM (in supporting information). In Fig. 2b,c, we also show the distributions of the electric field magnitude, for the reference and quasi-grating OSCs at the absorption peaks of the active layer (*λ* = 570 nm). With the presence of the quasi-grating structure, a significant increase in the near-field modal volume is evident, together with the enhancement of the electric field with extreme confinement in ultrathin active layer. The field distributions at the absorption peak of the total structure (*λ* = 670 nm) can be found in Figure S3a-b, exhibiting similar behavior.

**Figure 3:**
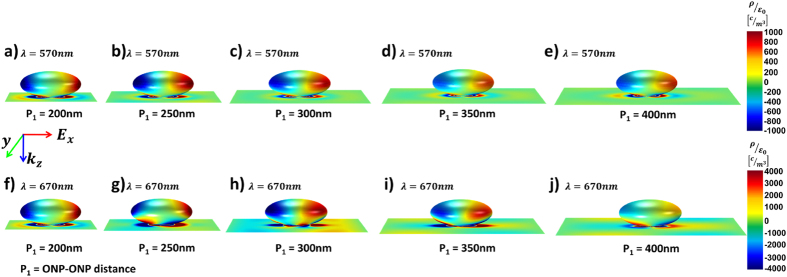
Induced surface charge distribution. Induced surface charge distribution of ONP array on the Ag substrate (anode) in the proposed OSCs as a function of the grating period at (**a–e**) *λ* = 570 nm and (**f–j**) *λ* = 670 nm.

Together with the induced surface charge distributions (see Fig. 3) calculated as a function of the grating period, the physical origins of the observed absorption enhancement can be better understood. Strong spatial-overlap between the LSP modes residing on the surface of the ONP and the plasmonic gap modes formed in the ONP-anode gap, provides robust mode-hybridization with negligible dependence on the grating period. It is worth noting that this strong mode hybridization around the ONP is physically different from the conventional simple combination of metal grating and metal nanoparticles located in different spatial regions (e.g., active layer and transport layer), which results in negligible mode-hybridization of spectrally-separated resonances^11^,^12^,^13^. As a result of strong mode hybridization in our case, dip-less wideband strong optical absorption becomes possible, tightly confined near the ONP-anode embedded in the ultra-thin active layer. Furthermore, this strong optical absorption limited to the near proximity of the ONP-anode region also yields the additional benefit of negligible device performance dependency on the period of the grating (Supplement Figure S3a,b), allowing large tolerances (190 nm ≤ *P*_*1*_ ≤ 400 nm) in the control of the ONP-ONP distance.

To test the device’s performance for all-day usage, the optical absorption under oblique incidence in the −70° < θ < 70° range also has been characterized. As a result of strong mode confinement near the ONP-anode substrate region, excellent optical absorption with an almost flat response across the incident angle variation of −60° < θ < 60° has been obtained (Figure S5a), irrespective of the incident angle (Figure S5b,c).

### Electrical characteristics: enhancement in hole collection from the quasi-grating anode

**Figure 4:**
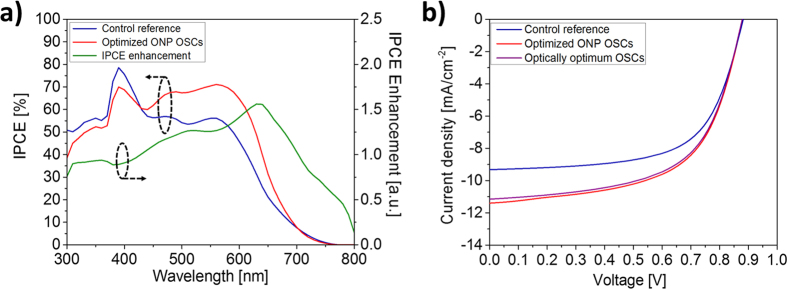
Electrical performance of the proposed quasi-grating OSCs. (**a**) Wavelength dependence of IPCE for control reference (blue) and quasi-grating inverted ultrathin OSCs (red), and IPCE enhancement (green). **(b)** J-V characteristics of control OSCs, optically-optimum OSCs and optimized quasi-grating OSCs. All data corresponds to AM 1.5G-weighted, normally-incident plane-wave illumination.

Having demonstrated the excellent light absorption properties of the proposed ONP quasi-grating OSCs, we now determine the actual device performance by calculating its electrical characteristics. First of all, to elucidate the impact of the dip-less wideband optical absorption on the electrical performance, we calculated the spectral response of incident photon to electron conversion efficiency (IPCE) and IPCE enhancement. The spectrum shown in Fig. 4a (blue line for the control device and red line for the quasi-grating OSCs) shows a clear enhancement in IPCE, with the introduction of the proposed ONP quasi-grating anode. Noting the spectral similarity between the calculated IPCE and optical absorption, these results indicate that there is a negligible penalty from the carrier recombination, with the reduced hole traveling lengths and the increased area of the hole-collection interface.

**Figure 5:**
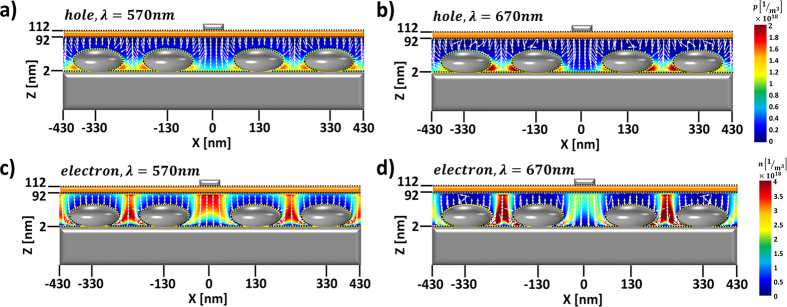
Carrier (hole and electron) density and current flow in the proposed quasi-grating OSCs, in the short-circuit condition. Hole concentration and hole current flow at (**a**) *λ* = 570 nm and (**b**) *λ* = 670 nm. Electron concentration with the current flow at (**c**) *λ* = 570 nm and (**d**) *λ* = 670 nm. The arrows denotes the amplitude and direction of the currents.

The details of the overall performance of the OSCs are summarized in Fig. 4b and Table 1. The performance factors of the control device are in good agreement with previous experimental reports^15^,^18^,^19^,^21^: with a power conversion efficiency (PCE) of 5.21%, an open-circuit voltage of 0.882 V, a short circuit current 9.32 mA/cm^2^, and a fill factor (FF; J_max_V_max_/J_sc_V_oc_) of 63.5%. To compare, for the proposed OSC, large improvements in J_sc_ and PCE were observed, approaching ∼22% and ∼15% respectively, with a PCE of 6.01% and an open-circuit voltage of 0.881 V, a short circuit current of 11.37 mA/cm^2^, and a FF of 60.0%. It is worthy of note that these performance factors that were achieved for the optical-electrical optimum design (at optical absorption efficiency of ~47%, with *r*_*x,y*_ = 70 nm, *r*_*z*_ = 30 nm ONPs of 200-nm period) are higher than those obtained from the optical-absorption optimum design (at optical absorption efficiency of ~49%, with *r*_*x,y*_ = 90 nm, *r*_*z*_ = 30 nm ONPs of 200-nm period), as summarized in Table 1. These observed performance differences are well explained by comparing the carrier transport, for the electrical-optical optimum (Fig. 5) and the optical-optimum design (Figure S6). Even though the increased eccentricity of the optically-optimal structure derives stronger mode hybridization for better optical properties, it is also observed that the same increased eccentricity of strong spatial mode localization induces non-uniform photo-carrier generation, perturbing the flow of carriers and thus reducing the power conversion efficiency and FF (see Table 1), in agreement with the prior art^7^,^20^,^25^,^26^.

## Discussion

In conclusion, we proposed a quasi-grating structure composed of an oblate nanoparticle array in electrical contact with the anode, for application to inverted ultrathin OSCs. Short-distance and efficient hole collection, together with dip-less strong hybridization in tight mode confinement provide large improvements in both PCE and broadband optical absorption when compared to the reference structure: ~33% in optical absorption, ~22% in the short circuit current, and ~15% in the PCE enhancements were obtained using coupled optical-electrical multidisciplinary numerical analysis. By analyzing the optical- and electrical-field profiles, together with surface charge distributions, exciton generation rates, IPCE spectra, J-V characteristics and the hole-electron concentration, the key factors and underlying physics for the observed device performance improvement have been clarified. The results of optical and electrical combined optimization for the device geometry clearly reveals the need for multi-domain design considerations involving both optical absorption and electrical carrier collection. The proposed platform of a quasi-grating structure offering strong plasmonic hybridization and increased anode surface area, together with the combined electrical-optical device optimization approach should be applicable to the design of highly efficient OSCs employing different active materials and optical-electrical scale lengths.

## Methods

### Comprehensive theoretical model

All simulations were performed using the finite element method (FEM) implemented in COMSOL Multiphysics^27^. Simulations consisted of two parts (the optical and electrical domains) sharing a common finite element mesh, generated by COMSOL Multiphysics. Optical parameters included complex refractive indices of PCDTBT:PC_70_BM, MoO3, and sol-gel processed ZnO measured by spectroscopic ellipsometry and the dielectric permittivity of Ag modeled using the measured data taken from Palik^28^. Electrical parameters included exciton lifetime (*τ*_*x*_) taken from refs 29 and 30, the diffusion coefficient (*D*_*x*_) taken from ref. 31, electron affinity (*χ*) taken from ref. 32, mean charge separation distance (*a*_*0*_), material related activation energy (*γ*) and exciton decay rate (*K*_*F*_) taken from ref. 31, electron and hole mobility (*μ*_*n*_, *μ*_*p*_) taken from refs 31 and 33, lifetime (*τ*_*n*_, *τ*_*p*_) taken from ref. 33, and band-gap and material energy levels taken from refs 22,34 and 35. In the optical simulations perfectly matched layers (PML) were used at the top and bottom unit cell boundaries, in addition to periodic boundary conditions between neighboring unit cells, under AM1.5G illumination conditions. Optical performance parameters of interest, such as the total absorption and the absorption in the active layer were calculated, defined as a fraction of the absorbed incident power (i.e., 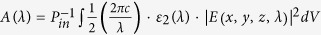). The electric field intensity *|E(x,y,z)|*^2^ was obtained via the optical simulation using the Maxwell equation solver and was then used as the input to the electrical part of the calculation, which solves the coupled-Poisson, charge carrier drift-diffusion and continuity equations. The coupled Poisson, carrier drift-diffusion and continuity equations are given by:


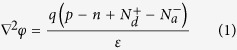



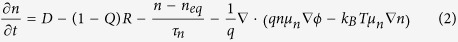



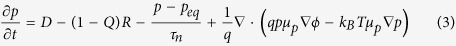


where *q* is the elementary charge, *k*_*B*_ is the Boltzmann constant, *ϕ* is the electrostatic potential, *Q* is the local dissociation probability, *R* is the local recombination rate, *μ*_*n*_ and *μ*_*p*_ are the electron and hole motilities, *N*_*d*_^+^ and *N*_*a*_^*−*^ are the concentrations of ionized donors and acceptors, *p* and *n* are the charge carrier densities, *p*_*eq*_ and *n*_*eq*_ are the equilibrium densities, and *τ*_*n*_, *τ*_*p*_ are the lifetimes of electrons and holes, respectively.

For the dissociation rate, we use the Onsager-Braun dissociation expressions (i.e., *D* = *G*_*opt*_*·Q(x,y,z)*) which include local changes in the exciton density due to field-dependent exciton dissociation probability. The exciton generation rate (in the active layer) was obtained from *G*_*opt*_*(λ)* = *ε“|E(x,y,z)|*^2^*/2ћ*, where *ε“* is the imaginary part of dielectric permittivity, and *ћ* is the reduced Planck constant (Figure S7). According to the Poole-Frenkel model the spatially-dependent electron or hole mobility is a function of the local electric field magnitude^36^,^37^: *μ(E,T)=μ*_*0*_*exp(γ((E*^*1/2*^*)/(T)))*, where *μ*_*0*_ is the zero-field mobility, *γ* is a material-related activation energy, and *T* is the temperature. J-V curves are then obtained by applying a potential difference between the top and bottom boundaries of the unit cell. From these J-V curves, we can calculate all standard solar cell performance parameters, such as J_sc_, V_oc_, FF, IPCE and PCE. We note that the band-to-band Langevin bimolecular recombination^38^, trap-assisted Shockley–Read–Hall (SRH) recombination^39^, surface recombination^40^ and the Onsager-Braun dissociation^26^,^41^,^42^,^43^ expressions are included in the continuity equations (Eq. 2, 3); deep trap states are not considered which is a common assumption in the modelling of organic solar cells. These states negligibly contribute to the space charge field^43^,^44^. Recently reported electrical effects, such as plasmonically induced hot carriers transfer and charge accumulation effects^15^,^45^ for the performance improvement of OSCs are not included in current analysis. More specific simulation details of the electrical simulation part such as determination of pre-illumination electronic equilibrium parameters can be found in 7, 22 and 40.

## Additional Information

**How to cite this article**: In, S. and Park, N. Inverted Ultrathin Organic Solar Cells with a Quasi-Grating Structure for Efficient Carrier Collection and Dip-less Visible Optical Absorption. *Sci. Rep.* **6**, 21784; doi: 10.1038/srep21784 (2016).

## Supplementary Material

Supplementary Information

## Figures and Tables

**Table 1:** Photovoltaic Performance Characteristics of OSCs under AM 1.5G-Weighted Illumination.

Case	*j*_sc_ (*m*A∕cm_2_)	V_*oc*_ (V )	FF (%)	*PCE* (%)
Control OSCs	9.32	0.882	63.5	5.22
Optically optimum ONP OSCs	11.15	0.88	59.5	5.84
Optimized ONP OSCs	11.37	0.881	60.0	6.01

## References

[b1] Tanaka Y., Sanada A. & Sasaki K. Nanoscale interference patterns of gap-mode multipolar plasmonic fields. Sci. Rep. 2, 764 (2012). 23097686 10.1038/srep00764 PMC3479447

[b2] Bouillard J. S. *et al.* Broadband and broadangle SPP antennas based on plasmonic crystals with linear chirp. Sci. Rep. 2, 829 (2012). 23170197 10.1038/srep00829 PMC3501754

[b3] Kollmann H. *et al.* Toward plasmonics with nanometer precision: nonlinear optics of helium-ion milled gold nanoantennas. Nano Lett. 14, 4778–4784 (2014). 25051422 10.1021/nl5019589

[b4] Jeong Y. G. *et al.* A vanadium dioxide metamaterial disengaged from insulator-to-metal transition. Nano Lett. 15, 6318–6323 (2015). 26352780 10.1021/acs.nanolett.5b02361

[b5] Chen X. *et al.* Atomic layer lithography of wafer-scale nanogap arrays for extreme confinement of electromagnetic waves. Nature commun. 4, 2361 (2013). 23999053 10.1038/ncomms3361

[b6] Chen H. H. *et al.* A plasmonic infrared photodetector with narrow bandwidth absorption. Appl. Phys. Lett. 105, 023109 (2014).

[b7] In S. *et al.* Enhanced light trapping and power conversion efficiency in ultrathin plasmonic organic solar cells: A coupled optical-electrical multiphysics study on the effect of nanoparticle geometry. ACS Photonics 2, 78–85 (2014).

[b8] Lee S., Mason D. R., In S. & Park N. Embedding metal electrodes in thick active layers for ITO-free plasmonic organic solar cells with improved performance. Opt. Express 22, A1145–A1152 (2014). 24978077 10.1364/OE.22.0A1145

[b9] Lee S., In S., Mason D. R. & Park N. Incorporation of nanovoids into metallic gratings for broadband plasmonic organic solar cells. Opt. Express 21, 4055–4060 (2013). 23481940 10.1364/OE.21.004055

[b10] Ameri T., Khoram P., Min J. & Brabec C. J. Organic ternary solar cells: a review. Adv. Mater. 25, 4245–4266 (2013). 23703861 10.1002/adma.201300623

[b11] Li X. *et al.* Dual plasmonic nanostructures for high performance inverted organic solar cells. Adv. Mater. 24, 3046–3052 (2012). 22566360 10.1002/adma.201200120

[b12] Li X. *et al.* High‐performance organic solar cells with broadband absorption enhancement and reliable reproducibility enabled by collective plasmonic effects. Adv. Opt. Mater. 3, 1220–1231 (2015)

[b13] You J. *et al.* Surface plasmon and scattering‐enhanced low‐nandgap polymer solar cell by a metal grating back electrode. Adv. Energy Mater. 2, 1203–1207, (2012).

[b14] Li X. H. *et al.* Efficient inverted polymer solar cells with directly patterned active layer and silver back grating. J. Phys. Chem. C 116, 7200–7206 (2012).

[b15] Petoukhoff C. E. *et al.* Plasmonic electrodes for bulk-heterojunction organic photovoltaics: a review. J. Photonics Energy 5, 057002–057002 (2015).

[b16] Cattin L. *et al.* MoO3 surface passivation of the transparent anode in organic solar cells using ultrathin films. J. Appl. Phys. 105, 4507 (2009).

[b17] Wu B., Wu Z., Tam H. L. & Zhu F. Contrary interfacial exciton dissociation at metal/organic interface in regular and reverse configuration organic solar cells. Appl. Phys. Lett. 105, 103302 (2014).

[b18] Jung K. *et al.* Plasmonic organic solar cells employing nanobump assembly via aerosol-derived nanoparticles. ACS nano 8, 2590–2601 (2014). 24533831 10.1021/nn500276n

[b19] Trost S. *et al.* Plasmonically sensitized metal-oxide electron extraction layers for organic solar cells. Sci. Rep. 5, 7765 (2015). 25592174 10.1038/srep07765 PMC4296292

[b20] Niesen B. *et al.* Plasmonic efficiency enhancement of high performance organic solar cells with a nanostructured rear electrode. Adv. Energy Mater. 3, 145–150 (2013).

[b21] Cowan S. R. *et al.* Chemically controlled reversible and irreversible extraction barriers via stable interface modification of zinc oxide electron collection layer in polycarbazole‐based organic solar cells. Adv. Func. Mater. 24, 4671–4680 (2014).

[b22] Sun Y. *et al.* Inverted polymer solar cells integrated with a low‐temperature‐annealed sol‐gel‐derived ZnO film as an electron transport layer. Adv. Mater. 23, 1679–1683 (2011). 21472797 10.1002/adma.201004301

[b23] Chu T. Y. *et al.* Highly efficient polycarbazole-based organic photovoltaic devices. Appl. Phys. Lett. 95, 63304 (2009).

[b24] Groep J. V. D., Spinelli P. & Polman A. Transparent conducting silver nanowire networks. Nano Lett. 12, 3138–3144 (2012). 22554260 10.1021/nl301045a

[b25] Smith A. J. *et al.* Repurposing Blu-ray movie discs as quasi-random nanoimprinting templates for photon management. Nature commun. 5, 5517 (2014). 25423591 10.1038/ncomms6517

[b26] Wei E. I., Choy W. C. & Chew W. C. The roles of metallic rectangular-grating and planar anodes in the photocarrier generation and transport of organic solar cells. Appl. Phys. Lett. 101, 223302 (2012).

[b27] Comsol Multiphysics, (2015) (date of access:28/12/2015). http://www.comsol.com.

[b28] Palik E. D. Handbook of Optical Constants of Solids. Academic Press: New York, 1985).

[b29] Etzold F. *et al.* Ultrafast exciton dissociation followed by nongeminate charge recombination in PCDTBT: PCBM photovoltaic blends. J. Am. Chem. Soc., 133, 9469–9479 (2011). 21553906 10.1021/ja201837e

[b30] Chen S. *et al.* Dielectric effect on the photovoltage loss in organic photovoltaic cells. Adv. Mater., 26, 6125–6131 (2014). 25070573 10.1002/adma.201401987

[b31] He Z. *et al.* Simultaneous enhancement of open‐circuit voltage, short‐circuit current density, and fill factor in polymer solar cells. Adv. Mater. 23, 4636–4643 (2011). 21905131 10.1002/adma.201103006

[b32] Hoke E. T. *et al.* The role of electron affinity in determining whether fullerenes catalyze or inhibit photooxidation of polymers for solar cells. Adv. Energy Mater. 2, 1351–1357 (2012).

[b33] Philippa B. *et al.* The impact of hot charge carrier mobility on photocurrent losses in polymer-based solar cells. Sci. Rep. 4, 5695 (2014). 25047086 10.1038/srep05695 PMC4105785

[b34] Chen Y., Lin W. C., Liu J. & Dai L. Graphene oxide-based carbon interconnecting layer for polymer tandem solar cells. Nano Lett. 14, 1467–1471 (2014). 24521516 10.1021/nl4046284

[b35] Shrotriya V. *et al.* Transition metal oxides as the buffer layer for polymer photovoltaic cells. Appl. Phys. Lett. 88, 073508 (2006).

[b36] Frenkel J. On pre-breakdown phenomena in insulators and electronic semi-conductors. Phys. Rev. 54, 647 (1938).

[b37] Melzer C., Koop E. J., Mihailetchi V. D. & Blom P. W. Hole transport in poly(phenylene vinylene)/ methanofullerene bulk-heterojunction solar cells. Adv. Funct. Mater. 14, 865−870 (2004).

[b38] Langevin P. Recombinaison et mobilites des ions dans ies gaz. Ann. Chim. Phys. 28, 433−530 (1903).

[b39] Shockley W. & Read W. T. Statistics of the recombination of holes and electrons. Phys. Rev. 87, 835 (1952).

[b40] Fonash S. Solar cell device physics. Elsevier, 2012).

[b41] Onsager L. Initial recombination of ions Phys. Rev. 54, 554–557 (1938).

[b42] Braun C. L. Electric-field assisted dissociation of charge-transfer states as a mechanism of photocarrier production J. Chem. Phys. 80, 4157–4161 (1984).

[b43] Wei E. I., Li X. & Choy W. C. Breaking the space charge limit in organic solar cells by a novel plasmonic-electrical concept. Sci. Rep. 4, 6236 (2014). 25168122 10.1038/srep06236 PMC4148652

[b44] Vervisch W. *et al.* Optical-electrical simulation of organic solar cells: excitonic modeling parameter influence on electrical characteristics. Appl. Phys. Lett. 98, 253306 (2011).

[b45] Choy W. C. & Ren X. Plasmon-Electrical Effects on Organic Solar Cells by Incorporation of Metal Nanostructures. IEEE J. Sel. Top. Quantum Electron. 22, 1–9 (2016).

