# Supplementary material for: Inverted Ultrathin Organic Solar Cells with a Quasi-Grating Structure for Efficient Carrier Collection and Dip-less Visible Optical Absorption
Source: Sci Rep. 2016 Feb 23;6:21784. doi: 10.1038/srep21784 (PMC4763232; doi:10.1038/srep21784)
Supplement: Supplementary Information [file srep21784-s1.doc]

Supporting Information:

Inverted Ultrathin Organic Solar Cells with a Quasi-Grating Structure for Efficient Carrier Collection and Dip-less Visible Optical Absorption

Sungjun In and Namkyoo Park*

Photonic Systems Laboratory, School of EECS, Seoul National University, Seoul 151-744, Korea

*nkpark@snu.ac.kr

**Fabrication method for quasi-grating structure.** The suggested spheroidal Quasi-Grating Structure (Figure 1a) could be fabricated by using the self-assembly processes^1^ or electron beam lithography^2^. First, a Si nanopillar array mold is prepared^1,2^ to construct a nanoporous PMMA imprint resist. Ag nanorod arrays are then selectively grown on the nanoporous templates with a Ag coated substrate. After the removal of PMMA, spheroidal quasi-grating structure can be obtained with the thermal annealing of Ag nanorod arrays^1-2^. It is noted that the period and geometry of the spheroid can be controlled with the process parameters used in the fabrication of Si array or annealing of Ag arrays.


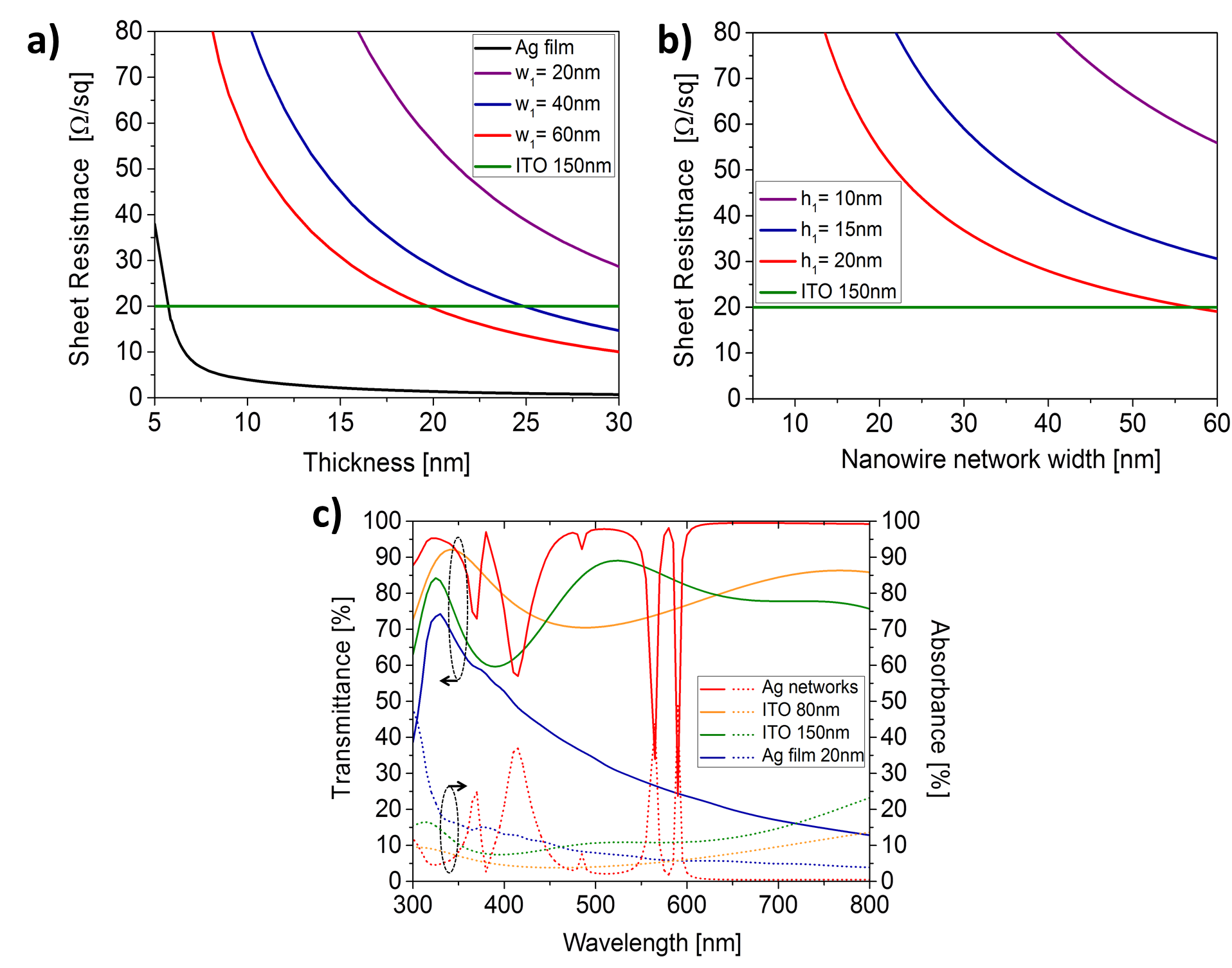


**Figure S1. Sheet resistances (*R_s_*) and optical transmittance *T(λ)* and Absorbance *A(λ)*.** Sheet resistance (*R_s_*) **(a)** as a function of film thickness for different electrodes: Ag film (black), various Ag nanowire network widths 20 (purple), 40 (blue) and 60 (red) nm, and reference 150 nm ITO film (green) **(b)** as a function of nanowire network width for various heights 10 (purple), 15 (blue) and 20 (red) nm, and reference 150 nm ITO film (green). **(c)** Spectral transmittance and absorbance of optimized Ag nanowire networks of 19.1 Ω/sq (red), 80 nm ITO of 58.2 Ω/sq (orange), 150 nm ITO of 20 Ω/sq (green), and 20nm thickness Ag film of 1.4 Ω/sq (blue).

**Electrical and optical characteristics of Ag nanowire networks electrode.** The sheet resistance (*R_s_*) of a Ag nanowire network is calculated (Eq. S1) to determine the nanowire dimensions, where *R_s_* is comparable to an ITO transparent electrode (uniform film) with the typical thickness of 150 nm^3-4^ and a measured Ag film electrode with the thickness of 5~30 nm^5^. We determined the *R_s_* of Ag nanowire networks by applying the Fuchs-Sondheimer(FS)-Mayadas-Shatzkes(MS) fitting model to include the quantum effect as follows^6-7^:

$R_{s}=\frac{\rho_{FS}+\rho_{MS}\text{-}\rho_{\text{bulk}}}{\text{w}_{1}}\times\frac{Period}{h_{1}}$ (S1)

Where *ρ_FS_*, *ρ_MS_* and *ρ_Bulk_* are the resistivity (in the units of *Ωm*) of a thin metal film from the FS model, MS model, and bulk metal, respectively. Figure S1a shows *R_s_* as a function of film thickness with period of (800 + width) nm and Figure S1b shows *R_s_* as a function of the Ag nanowire network width with a fixed period of 860 nm; *R_s_* is inversely proportional to thickness and width. Calculated sheet resistance of Ag nanowire network, used in our study ranges from 10~80 Ω/sq, and is in good agreement with previous experimental reports^8-9^. To ensure a better sheet resistance than the practical 80~150 nm ITO film (*R_s_*=20 Ω/sq^10^), it is evident that metal electrodes with 60 nm width should be chosen when thickness is less than or equal to 20 nm with a period 860 nm. Figure S1c shows transmittance and absorbance of different electrodes. Networks of Ag nanowire are made with a height of 20 nm, width of 60 nm and pitch of 860 nm for sufficient light transmission (average transmission of 91.7%), and also to guarantee low sheet resistance (19.1 Ω/sq). For the conventional ITO electrodes (80~150 nm thickness), normally the averaged transmission is around 77~80% and for Ag thin film electrodes, around 33%.


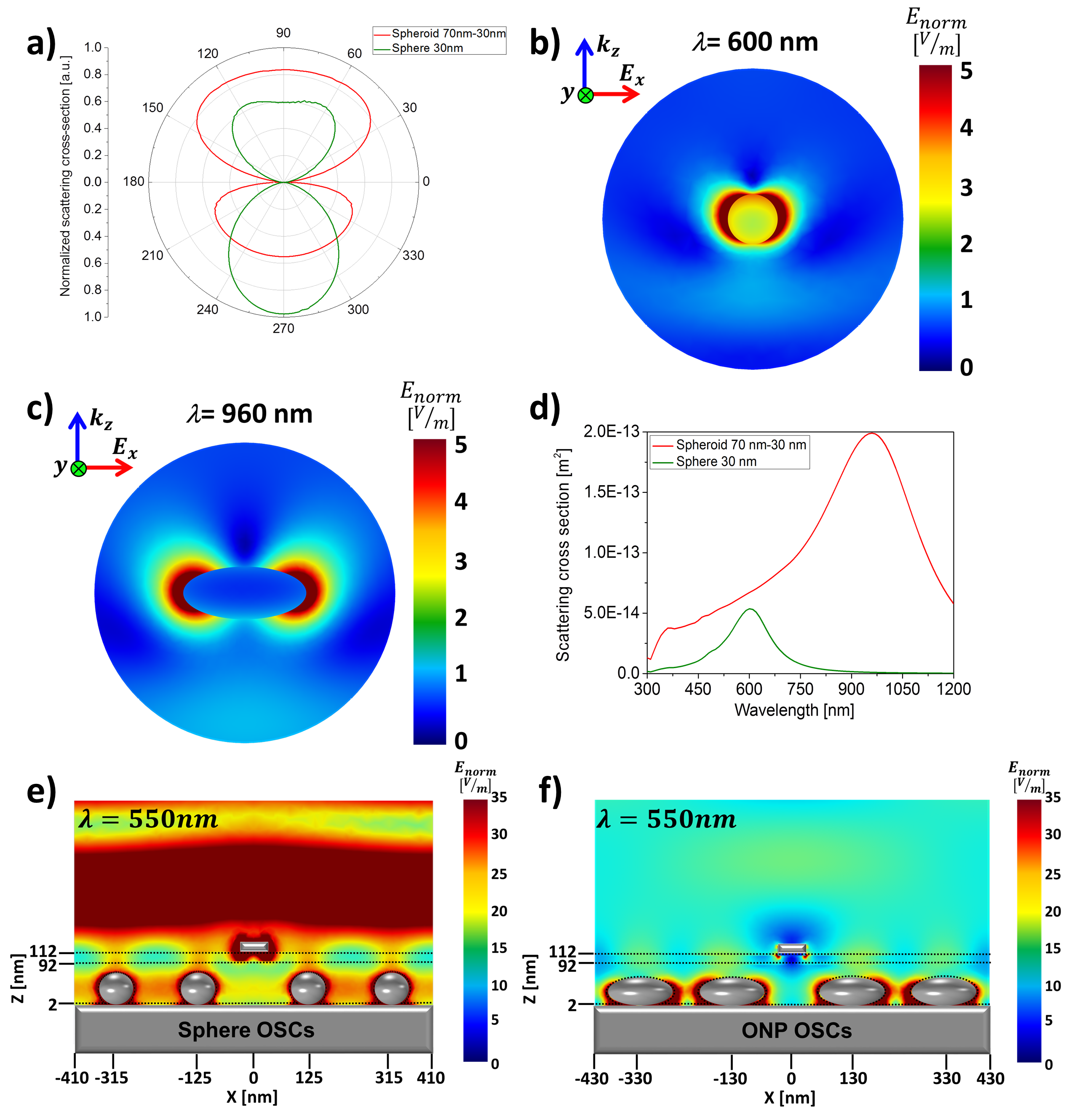


**Figure S2.** Scattering intensity polar plot **(a),** electric field distributions of an isolated **(b)** NP sphere and **(c)** ONP immersed in a uniform medium with refractive index n = 2.08 (the average index of PCDTBT:PC_70_BM in the considered wavelength range 300 nm ≤ λ ≤ 800 nm) and scattering cross-section spectra **(d).** Calculated electric field distributions (|E|) of **(e)** sphere OSCs, and **(f)** optimized ONP case at λ = 550 nm.

Figure S2a compares the in-plane scattering of isolated ONP to that of SNP, assuming a surrounding medium of effective n = 2.08, obtained for PCDTBT:PC_70_BM in the interested wavelength range. It is worth to mention that, when ONPs are deposited on top of an OSC anode, the strong hybridization between the ONP LSP mode and gap mode leads to a blue shift of the resonance frequency to visible (Figure 2a), meanwhile its strong in-plane scattering features retained (Figure S2e-f).
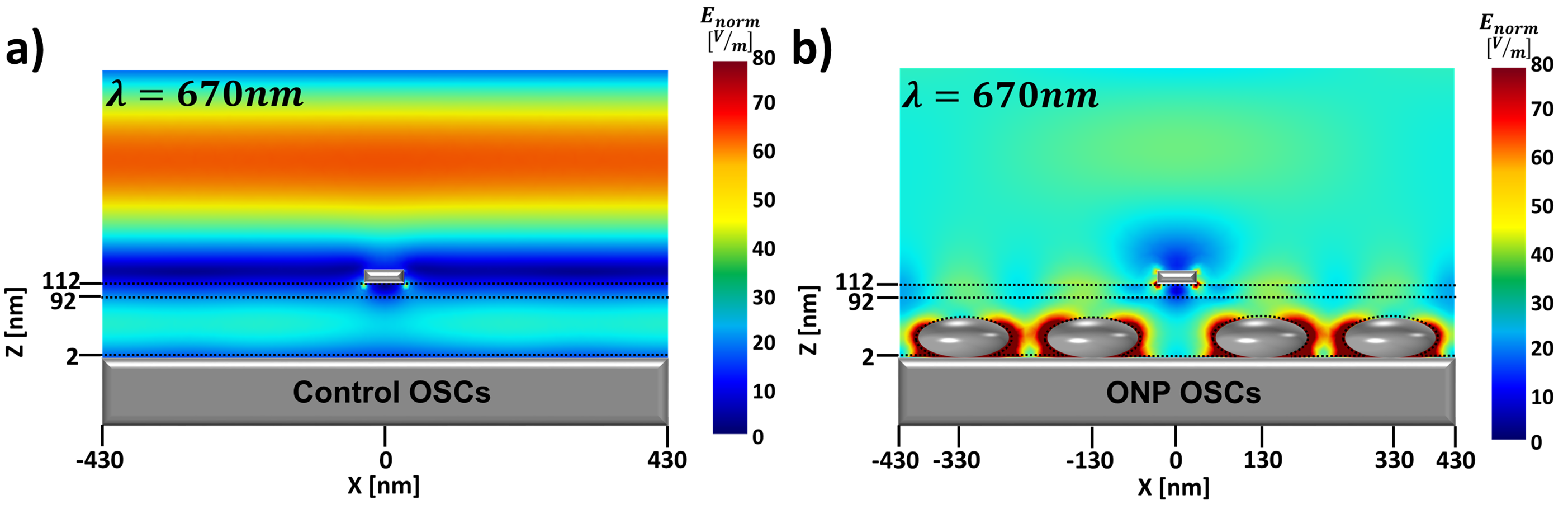


**Figure S3.** Electric field (magnitude) distributions, similar to Figure 2 **(b-c)** except with *λ* =670 nm.


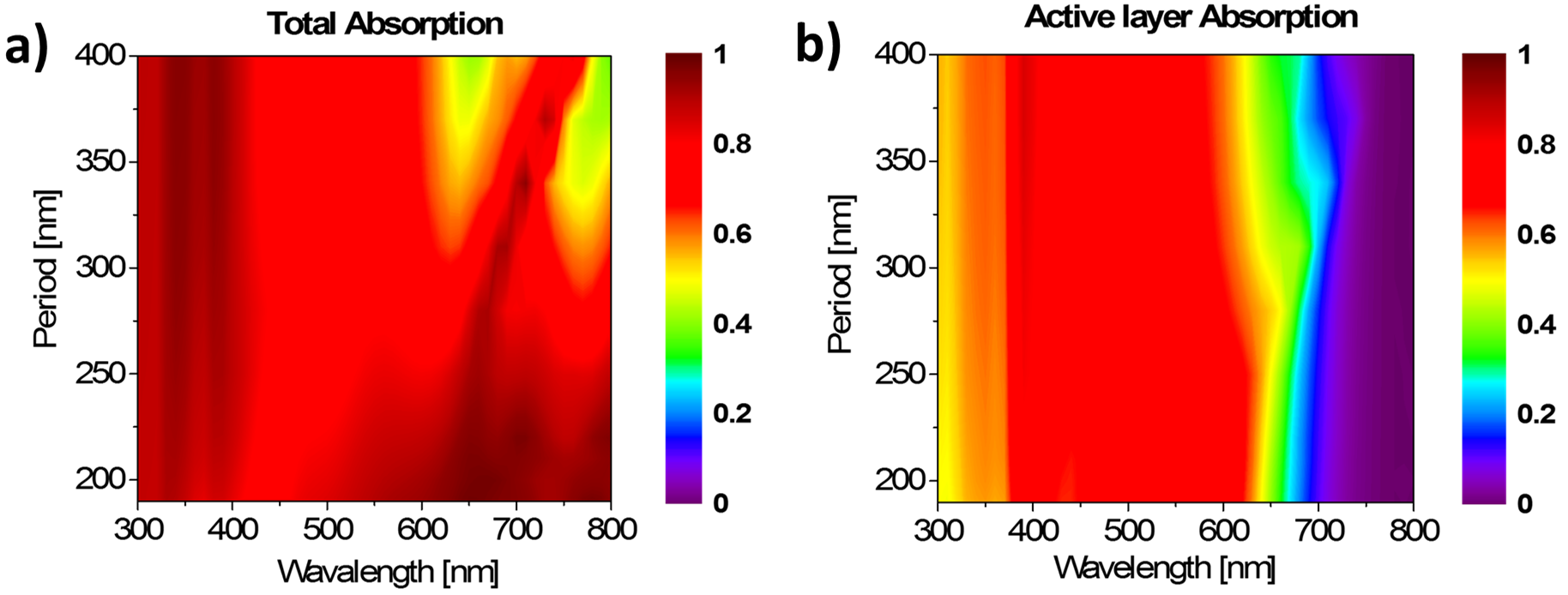


**Figure S4.** Absorption maps with respect to the ONP array period P_1_ for **(a)** total structure and **(b)** active layer.

**Definition of a figure of merit (FOM).** Optical absorption capability of the ONP quasi-grating inverted ultrathin OSCs is characterized by the figure of merit, defined as the percentage of incident photons (with AM1.5G spectral weighting) that are absorbed in the active layer:

$$FOM=\left(\int_{\lambda_{300}}^{\lambda_{800}} \frac{\lambda}{hc}\cdot I\left(\lambda\right)\cdot A\left(\lambda\right)\cdot d\lambda\right)/\left(\int_{\lambda_{300}}^{\lambda_{800}} \frac{\lambda}{hc}\cdot I\left(\lambda\right)\cdot d\lambda\right)$$

Where *h* is the Plank constant, *c* is the speed of light in free space, *I(λ)* is the AM1.5G solar spectrum weighting factor, *A(λ)* is the optical absorption as in Figure 2a, and the integration region is the active layer excluding the ONP array.


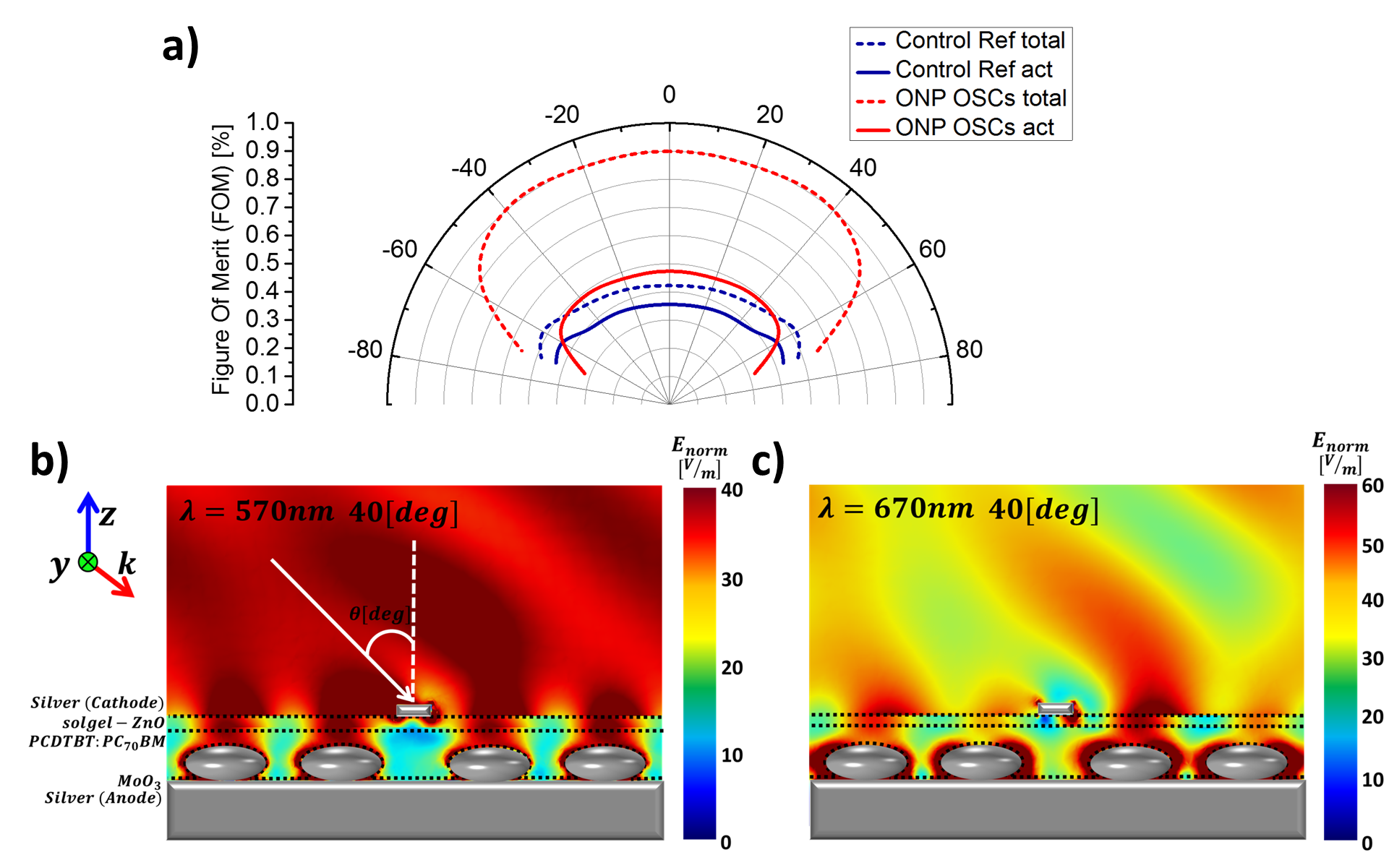


**Figure S5. Dependence of optical absorption on incidence angle. (a)** Incidence angle dependence of the FOM for the control reference (blue lines) and quasi-grating inverted ultrathin (red lines) OSCs. Calculated electric field distributions (|E|) of the optimized ONP case **(b)** at *λ* = 570 nm, 40°, **(c)** at *λ* = 670 nm, 40°.


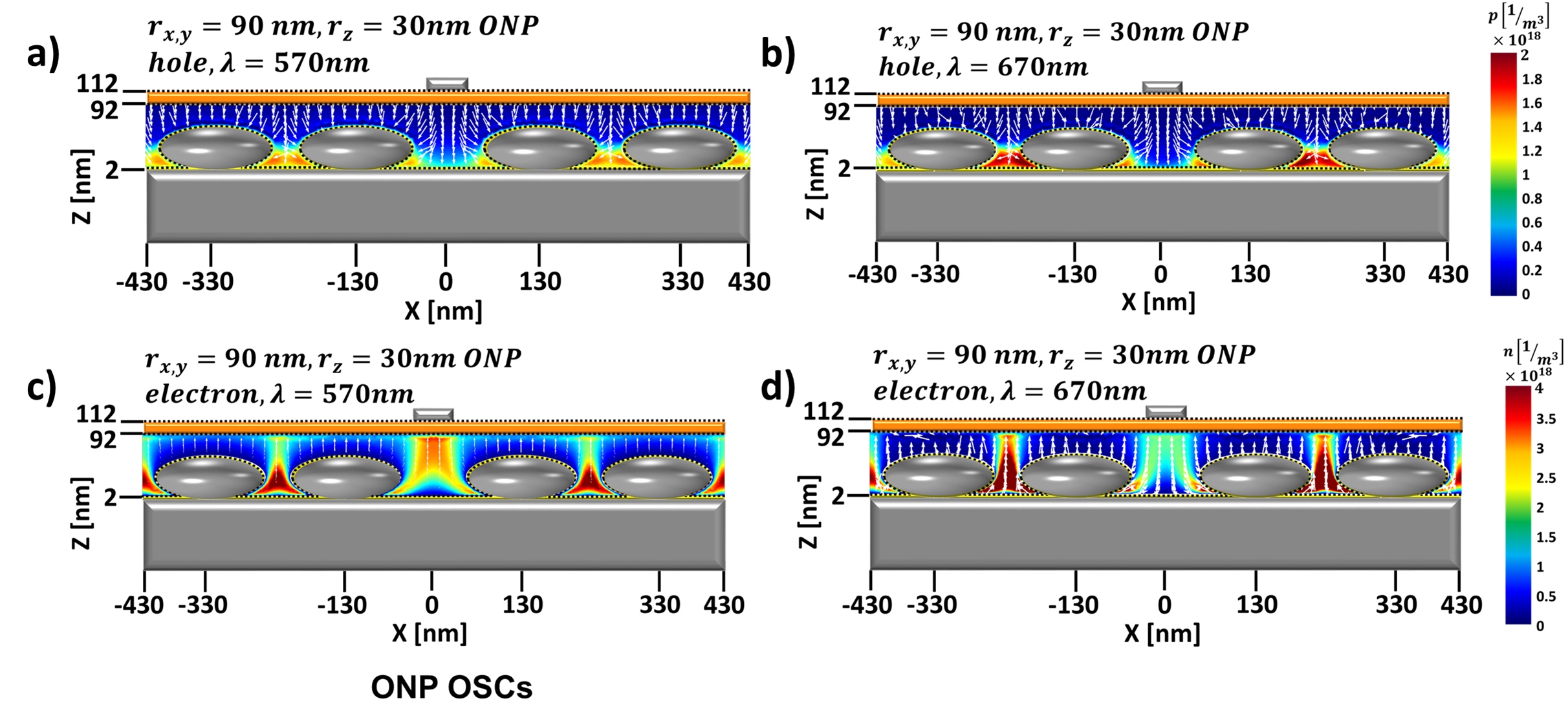


**Figure S6. Carrier (hole and electron) density and current flow in the optically-optimum quasi-grating OSCs, in the short-circuit condition.** Hole concentration and the hole current flow at **(a)** *λ* = 570 nm and **(b)** *λ* = 670 nm. Electron concentration with the electron current flow at **(c)** *λ* = 570 nm and **(d)** *λ* = 670 nm. The arrow denotes the amplitude and direction of the currents.


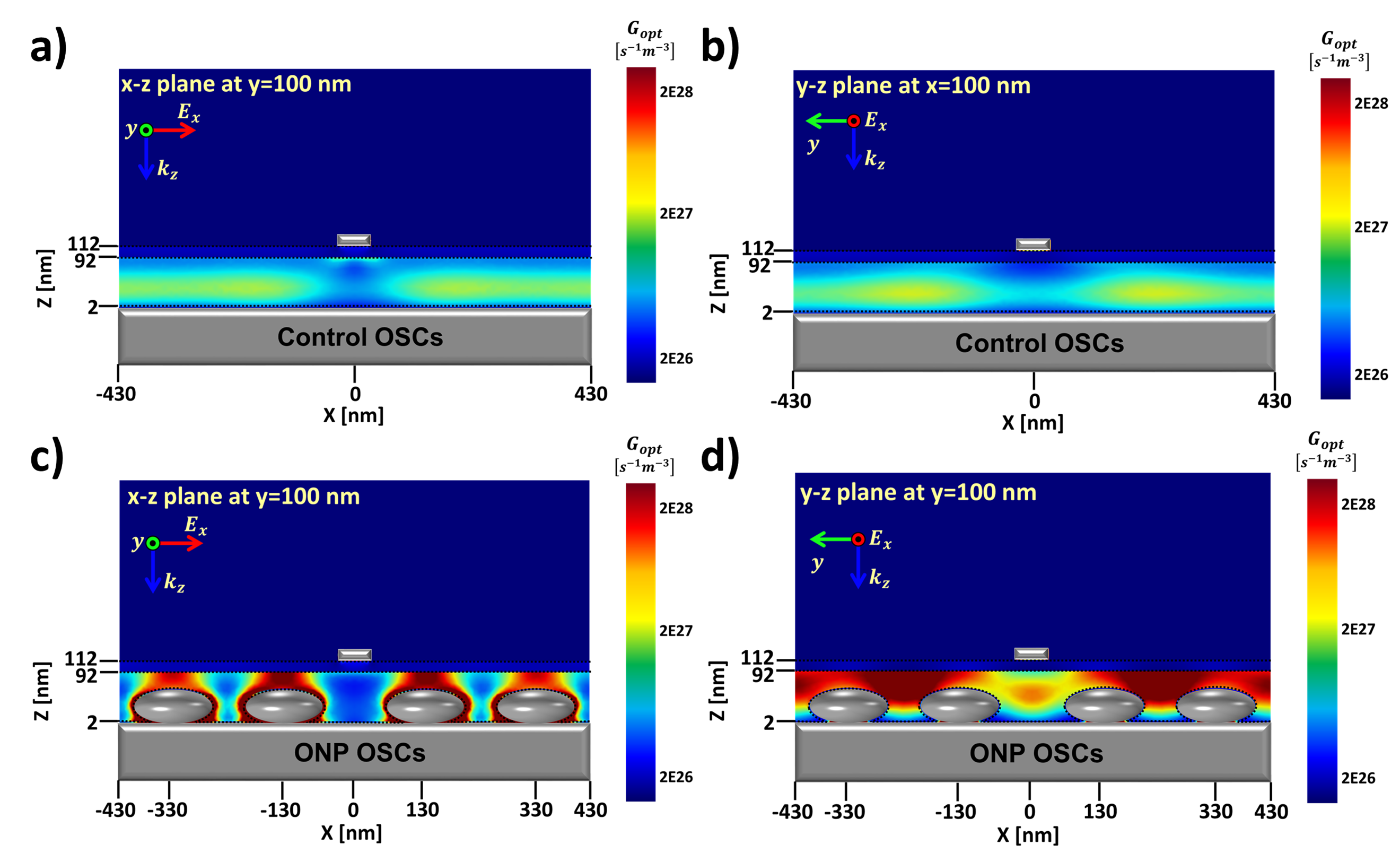


**Figure S7.** Total exciton generation rates of **(a-b)** control OSCs and **(c-d)** ONP quasi-grating inverted ultrathin OSCs under AM 1.5G-weighted, normally-incident plane-wave illumination (describing the *x-z* plane at *y* = 100 nm and the *y-z* plane at *x* = 100 nm).

**References**

1. Krishnamoorthy, S., Krishnan, S., Thoniyot, P., Low, H. Y. Inherently reproducible fabrication of plasmonic nanoparticle arrays for SERS by combining nanoimprint and copolymer lithography. ACS appl. Mater. Interfaces 3, 1033-1040 (2011).
2. Spinelli, P., Hebbink, M., De Waele, R., Black, L., Lenzmann, F., Polman, A. Optical impedance matching using coupled plasmonic nanoparticle arrays. Nano Lett. 11(4), 1760-1765 (2011)..
3. Wang, D. H., Kyaw, A. K. K., Pouliot, J. R., Leclerc, M., Heeger, A. J. Enhanced Power Conversion Efficiency of Low Band‐Gap Polymer Solar Cells by Insertion of Optimized Binary Processing Additives. Adv. Energy Mat. 4 (2014).
4. Chu, T. Y., Alem, S., Verly, P. G., Wakim, S., Lu, J., Tao, Y., Serge B., Mario L., Francis B., Denis D., Sheila R., David W., Gaudiana, R. Highly efficient polycarbazole-based organic photovoltaic devices. Appl. Phys. Lett. 95, 63304 (2009).
5. Romanyuk, A., Steiner, R., Mack, I., Oelhafen, P., Mathys, D. Growth of thin silver films on silicon oxide pretreated by low temperature argon plasma. Surf. sci. 601, 1026-1030 (2007).
6. Lee, S., Mason, D. R., In, S. Park, N. Embedding metal electrodes in thick active layers for ITO-free plasmonic organic solar cells with improved performance. Opt. Express 22, A1145-A1152 (2014).
7. O’Connor, B., Haughn, C., An, K. H., Pipe, K. P., Shtein, M. Transparent and conductive electrodes based on unpatterned, thin metal films. Appl. Phys. Lett. 93, 223304 (2008).
8. van de Groep, J., Spinelli, P., Polman, A. Transparent conducting silver nanowire networks. Nano Lett. 12(6), 3138-3144 (2012).
9. Lee, J. Y., Connor, S. T., Cui, Y., Peumans, P. Solution-processed metal nanowire mesh transparent electrodes. Nano Lett. 8(2), 689-692 (2008).
10. Jung, K., Song, H. J., Lee, G., Ko, Y., Ahn, K., Choi, H., Kim, J. Y., Ha, K., Song, J., Lee, J. Lee, C. & Choi, M. Plasmonic organic solar cells employing nanobump assembly via aerosol-derived nanoparticles. ACS nano 8, 2590-2601 (2014).
